# Cumulative Effect and Content Variation of Toxic Trace Elements in Human Hair around Xiaoqinling Gold Mining Area, Northwestern China

**DOI:** 10.3390/ijerph18042074

**Published:** 2021-02-20

**Authors:** Min Yang, Youning Xu, Hailing Ke, Huaqing Chen

**Affiliations:** 1 School of Resources Engineering, Xi’an University of Architecture & Technology, Xi’an 710055, China; 2 Shaanxi Tongguan Observation Base on Geological Environment of Mines, Xi’an Center of China Geological Survey, Xi’an 710054, China; xyouning@cgs.cn (Y.X.); khailing@cgs.cn (H.K.)

**Keywords:** toxic element accumulation, human hair, gold mining area, Xiaoqinling Mountains

## Abstract

The harm of toxic trace element polluted living environments to human health in mining areas has attracted extensive attention. In this study, human hair samples from a toxic trace element polluted area (village A) in a mineral processing area collected in 2015 and 2019 were studied in detail and the nonpolluted human hair samples from a contrast area (village B) with a relatively clean environment were also collected for comparison. The Hg and As in human hair samples were analyzed by Atomic Fluorescence Spectrometry (AFS) and the Pb, Cd, Cr, and Cu in human hair samples were analyzed by Inductively Coupled Plasma Mass Spectrometry (ICP-MS). The single cumulative index (P_i_) and the Nemerrow index (P_z_) were used to evaluate the single and comprehensive cumulative pollution index. The results indicated that the average toxic trace element contents in human hair from different ages in the polluted area exhibited certain statistical significance. The average single cumulative indexes indicated a significant accumulation of Hg, Pb, and Cd in human hair of both genders and different ages from the polluted area, and the comprehensive cumulative pollution indexes revealed higher accumulation of toxic trace elements in the hair of males than in females. In general, the content of toxic trace elements in human hair from polluted area was still growing in accumulation. The high content of toxic trace elements in human hair shows a notable correlation with human health, and the environmental pollution in gold mining areas is seriously harmful to human health.

## 1. Introduction

Heavy metals and toxic trace elements, especially Hg, Cd, Pb, Cr, As, and Zn, have significant biological toxicity. They cannot be degraded by microorganisms in soil and water environments but can only be transformed, dispersed, and enriched between various forms. Occupational exposure or long-term, low-level exposure will lead to Minamata disease (Hg), Itai-itai disease (Cd), arsenic poisoning (As), etc., which can cause body malformation and even endanger life. The toxic trace elements in the human bodies are mainly englobed by daily drinking, breathing, eating, and will be harmful to human health when beyond a certain amount. The mining of mercury, lead-zinc, copper, gold, nickel, chromite and other metal mineral resources will expose the deep buried ores to the Earth’s surface. The main components and symbiotic or associated toxic elements will leach with mining gangue and drain into water and soil environments. More importantly, a very small part of Hg, Pb, Zn, Cu, Cd, As, and Cr will be diffused through industrial fumes, tailings slurry, and waste gangue in the process of beneficiation and metallurgy causing environmental pollution in rivers, groundwater, air, and soil and endangering people’s health directly or indirectly. Activities of mineral mining have led to serious pollution of fertile farmlands in many developing countries including some poor and backward areas of western China and were caused by weak awareness of environmental protection, loose administration, and the single pursuit of economic development [1]. In the past three decades of rapid economic development in China, only a few local governments have issued environmental protection regulations and set protecting financial funds in standardizing mining order, restoration of mining environment, and eliminating outdated technology involving low-tech, labor-intensive mining and mineral processing [2,3,4,5,6,7,8,9,10]. This choice has suppressed the development of China’s mining economy in the short term. However, through the reform and industrial upgrading of large state-owned enterprises, the mining economy is gradually picking up [11,12].

Human hair is an effective indicator to evaluate human exposure to some toxic elements. The World Health Organization (WHO), the U.S. National Environmental Protection Agency (EPA), and the International Atomic Energy Agency (IAEA) declared that elements in human hair are accurately determined and represent the overall level of body elements [13]. It is usually used for heavy metal exposure assessment, regional biological death monitoring, retrospective investigation, and medical diagnosis [14]. In clinical applications of hair analysis, the hair elemental contents and their relative ratios have certain practical values and implications [15]. As a recognized biological marker for human health monitoring, modern medicine has taken the contents of Pb, Cr, Cd, and As in human hair as the detection indicators of environmental pollution caused by occupational poisoning [16]. In developing nations, inhabitants of settlements around active and abandoned mineral mining areas are exposed to toxic trace elements emanating from mineral exploitation and processing. These harmful trace elements have contaminated soils and rivers in China [17], Ecuador [18,19], Nigeria [20,21], and Ghana [22,23]. These pollution incidences have led to many health issues in the areas where the mining and mineral processing activities are carried out [24]. At present, many scholars have studied the trace elements in human hair. The heavy metals Sb, As, and Hg in antimony mining and smelting areas are significantly more harmful to human health than the corresponding heavy metals in non-antimony mining areas [25]. Some researchers reported a more serious harm due to Pb and Cd contents to juvenile group (under 15 years old) with more significant amount of Pb and Cd elements in juveniles’ hair, and reliable correlations were established among the contents of Cu, Zn, Pb, Cd, and As in human hair around the Dexing mining area [26]. The distribution of toxic trace elements in human body is controlled by the human living environment. The content of toxic trace elements in human body is closely related to the geological background. The source of toxic trace elements’ concentration in human hair is highly related to the small-scale crude metal smelting process. The content of contaminating elements was relatively high in human bodies living in the metal processing industrial area, where 33% of the samples showed high As content beyond healthy control limits. The Cu content in the water of the Le’an River flowing through Dexing Copper Mine in Jiangxi Province is positively correlated with the Cu content in children’s hair along the river. The trace element content in children’s hair is consistent with the pollution status and the regularity of pollutant reduction in the river [27]. The Xiaoqinling gold mining area is a typical mining area in China where soils and some rivers were seriously polluted by Hg, Pb, and Cd [28,29,30,31,32,33,34,35,36,37]. The three main objectives of the present study are as follows: (1) to investigate the concentration of six toxic trace elements in human hair in Xiaoqinling gold mining area; (2) to evaluate age- and gender-related variations in levels of toxic metals; and (3) to estimate the temporal effects by comparing toxic metal results in human hair from 2015 to 2019 in the same village with severe toxic element pollution; (4) to estimate the spatial effects by comparing toxic metal contents in human hair from the polluted area and the contrast area. Some recent studies usually compare the results of the polluted area with other polluted or nonpolluted areas in published literature while this study selected a contrast area with the same geological environment [38]. This kind of contrast area can prevent the influences of different backgrounds in the study. The findings may provide a scientific basis for early warning of environmental pollution in mining and mineral processing areas.

## 2. Materials and Methods

### 2.1. Description of the Xiaoqiinling Gold Mining Area

**Figure 1:**
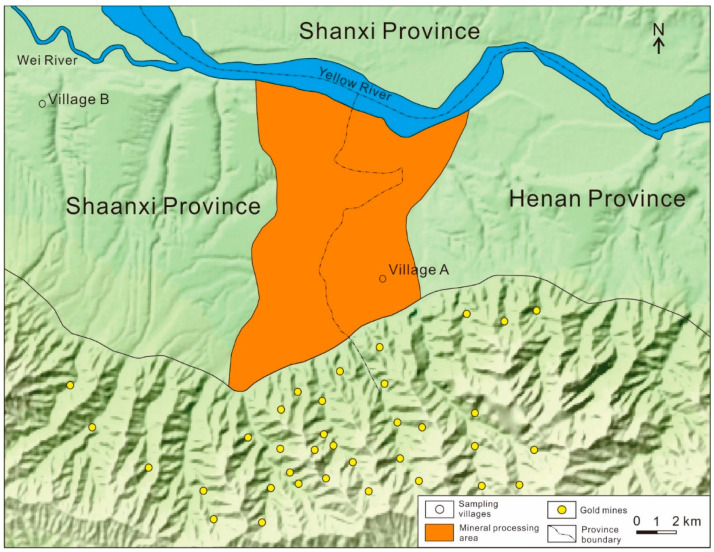
The map of the study area.

The Xiaoqinling gold mine belt spans Henan and Shaanxi provinces from east to west including Lingbao County, Tongguan County, Luonan County, and Huayin County. The areas of gold mining, smelting, and processing are near 1500 km^2^, which is the second largest hard-rock gold mining and processing base of China after Zhaoyuan gold mine in Shandong Province. The gold mining area is mainly distributed in the Xiaoqinling Mountains, and the agricultural area with many mineral processing and smelting industries is located especially at the junction region of Shaanxi and Henan province (Figure 1). Due to historical reasons, Xiaoqinling gold mining area is a typical representative of China’s disorder and weak awareness of environmental protection in mining development from 1986 to 2000. There were hundreds of large and small corporations and individually owned workshops in mining and beneficiation. The town- and individually owned workshops mainly used crude flotation, amalgamation grinding, cyanidation pond, and other methods for gold extraction. Crude flotation, amalgamation grinding, and cyanidation pond were widespread around the Xiaoqinling gold mining area and mainly adopted by local village collectives and individuals for gold extraction. The waste slag of mineral processing and smelting is dumped randomly in the fields, around the villages, and along the roads and rivers. Rainfall leaching, atmospheric dust fall, and river scour accelerate toxic trace elements entering rivers and soil, causing serious Hg, Pb, Cd, and Cu pollution in the soil and water environment around the mining areas. Some literature on the Xiaoqinlin gold mining area indicated the Hg in the soils and rivers mainly comes from the mercury flotation beneficiation. The Pb, Cu, Cd, and Zn are mainly provided by galena, sphalerite, chalcopyrite, and other ore minerals in gold-bearing quartz veins, while As and Cr exhibit poor correlation with Pb, Cu, Cd, and Zn [34].

### 2.2. Sample Collection and Preparation

In order to study the impact of toxic trace element contents in the environment on human health, the spatial and temporal comparison methods were applied. The accumulation of toxic trace elements in human hair can be assessed by comparing the content of toxic trace elements in human hair from polluted areas and contrast areas. Although an effective evaluation standard for the accumulation of toxic trace elements in human hair was not established, the average content of toxic trace elements in human hair from the contrast area under the same geological environment background, the same dietary habits, without artificial environmental pollution, and beside the polluted area could be reasonably used as a comparison to evaluate the accumulation with the aim of reflecting the impact of toxic trace element pollution on human health.

In this paper, the human hair from village A with serious cross-contamination of water and soil environment was collected and analyzed. Within 1 km from village A, a long history of gold ore processing and smelting activities has been ongoing for near three decades. Some villagers are engaged in small cyanide, small mercury mixing mill, gold burning, and mercury discharging activities in their own courtyard or in front and behind their houses. At present, there are still sporadic gold extraction activities around the village. Twenty and sixty-five human hair samples were collected from both genders and different ages in 2015 and 2019, respectively. Village B, which is located 15 km away from the village A, was selected as the contrast area because of the weak mineral and metallurgical activities in history and its clean water and soil environment. The hair samples from village B were randomly collected from four and seventeen volunteers of both genders and different ages in 2015 and 2019. The two sampling years included the same eleven volunteers, nine in village A and two in village B, expecting to obtain the temporal changes of toxic trace element contents in human hair. The residential environmental situation and the relationship between the health status of the population and the pollution sources were also investigated and recorded during the sample collection.

Two grams of hair were cut from the back of the head of each person, and stored in plastic bags. The samples were processed and analyzed by the Laboratory of the Northwestern Supervision and Monitoring Center for Mineral Resources, the China Geological Survey. Hair samples were soaked in absolute ethanol for 1 h in the laboratory, then poured out ethanol and soaked in 75% ethanol for 1 h, then washed with distilled water and dried naturally. Next, 1 g of hair samples was weighed and placed in a beaker. The samples were digested with nitric acid and left overnight. After heating the samples until almost dry, and the samples were repeatedly processed with permanganic acid until the yellow color faded, then 5 mL nitric acid was added, the volume was set to 25 mL with distilled water, and permanganic acid was added to smoke.

### 2.3. Pollution and Accumulation Evaluation

In this study, the cumulative degree of toxic trace elements in human hair was described by single pollution index and comprehensive pollution index. The higher content in human hair than in the control area was considered as the result of environmental toxic elements’ effect.

The single cumulative index of toxic trace elements in human hair is showed in following equation [39]:

(1) $$P_{i}=\frac{C_{i}}{C_{i0}}$$

where *P_i_* is the single cumulative index of toxic trace elements in human hair. The higher the *P_i_* value, the higher the accumulation of toxic trace elements in the human hair compared with the control area, and the greater the risk of people suffering from toxic trace element hazards. *C_i_* is the content of i-typed toxic trace element in human hair of village A in contaminated area (mg/kg); *C_i_*_0_ is the average content of i-typed toxic trace element in human hair of village B in the contrast area (mg/kg).

The Nemerrow index was used to evaluate the compound accumulation degree of toxic trace elements in the human hair of village A in the polluted area, and is also known as the comprehensive cumulative pollution index [40,41]:

(2) $$P_{z}=\sqrt{\frac{{\left(maxP_{i}\right)}^{2}+{\left(\bar{P_{i}}\right)}^{2}}{2}}$$

where *P_Z_* is the comprehensive cumulative pollution index of toxic trace elements in human hair; *P_i_* is the single cumulative pollution index of toxic trace elements; max *P_i_* is the largest single accumulative pollution index value of the six toxic elements; $\bar{P_{i}}$ is the average of the single accumulative pollution index of the six toxic elements.

The cumulative rate of toxic trace elements (*R_a_*) in human hair was determined by the ratio of the number of samples with toxic trace element contents higher than the contrast area (*N_a_*) and the total number of samples in the polluted area (*N_t_*):

(3) $$R_{a}=\frac{N_{a}}{N_{t}}\times 100\%$$

The cumulative contribution rate of type i toxic trace element (*R_c_*) in human hair was determined by the ratio of the single pollution index of type i toxic trace element (*P_i_*) and the sum of the single pollution index of all types of toxic trace elements.

(4) $$R_{c}=\frac{P_{i}}{\sum_{i=1}^{n}P_{i}}\times 100\%$$

where *n* is the number of total samples.

The growth rate (*R_g_*) of the toxic trace elements in the eleven volunteers sampled in both 2015 and 2019 was calculated by dividing the numbers of the toxic trace element increased from 2015 to 2019 (*N_g_*) into the total sample number (*N*):

(5) $$R_{g}=\frac{N_{g}}{N}\times 100\%$$

### 2.4. Analytical Methods

The analytical instruments for determining the toxic trace element contents in the human hair samples were provided by the Xi’an Center of China Geological Survey. Hg and As were determined by Atomic Fluorescence Spectrometer (Haiguang AFS 9760) and Pb, Cd, Cr, and Cu were analyzed by Inductively Coupled Plasma Mass Spectrometry (Agilent 7700x ICP-MS). A certified human hair reference material (GBW07601a, National Research Center for Certified Reference Materials, Beijing, China) was used to ensure the accuracy of the analytical data, and the average relative standard deviation for instrumentation was less than 5%.

## 3. Results

### 3.1. Content Characteristics of Toxic Trace Elements in Human Hair

Eighteen was used as the distinguishing age between adults and juveniles. As shown in Table 1, the average contents of toxic trace elements in the hair of the four categories, namely, adult male, adult female, juvenile male, and juvenile female in the contrast area were coincident. Only the Pb in the hair of juvenile males and the Hg, Pb, and Cd in the hair of juvenile females were higher than those of other groups. Moreover, the Pb content in the hair from the juvenile group was significantly higher than that of the adults. In addition, only the contents of Pb in the human hair samples from the contrast area were obviously higher than the average Pb result in the Chinese resident normal hair content, which indicates a relatively high background of Pb in Xiaoqinling Mountains.

Table 2 showed a prominent statistical significance in the average content of toxic trace elements in the hair of 65 volunteers in the polluted area. The Hg in the hair samples of the volunteers aged from 40 to 49 years old was the highest (3.492 mg/kg), followed by people aged between 60 and 77 years old (which was associated with disordered gold mining and leaching activities in this area during 1986 to 2000, and these villagers participated in gold mining and mineral processing in the period) and the children aged from 2 to 9 years old. However, the elements Pb, Cd, As, and Cu in the hair of children aged from 2 to 9 years old were significantly higher than those in other age groups. Therefore, the accumulated characteristics of toxic trace elements in the hair of children under the age of 9 were obviously observed, and they were the most susceptible individuals suffered from environmental toxic trace element pollution and health risk.

As shown in Table 3, Hg, Pb, Cd, Cr, As, and Cu could be detected in the human hair of different groups in both polluted and contrast areas. The average contents of Hg, Pb, and Cd in hair of different people in the contaminated area were higher than those in the contrast area. Except for As element in adult women’s hair, the As and Cu content in the other groups was higher than that in the contrast area, and the Cr content in all the samples were lower than that in the contrast group. Adult males accumulated the most Hg, Pb, and Cd, followed by juvenile males. In general, the toxic trace element content in male hair is higher than that in female hair, while the toxic trace element content in adult hair were higher than that in same-sex juveniles. Therefore, environmental pollution caused by Hg, Pb, Cd, and As elements has become the dominant hazard endangering people’s health.

The contents of Pb element in the hair of two girls aged two years old from the polluted area were 368 and 574 mg/kg, respectively, which were 12.71 and 19.82 times higher than the average Pb content of human hair (28.95 mg/kg) in the contrast area. This phenomenon indicated that children bodies are more likely to accumulate Pb element and are in the highest risk. In some previous studies, the general trend of Pb in human hair showed a regular decrease with increase in age [42], and the environmental pollution is harmful to juveniles.

### 3.2. Analysis of Toxic Trace Element Accumulation in Human Hair

From Table 4, for both genders and different ages in the polluted area, Hg, Pb, and Cd were the most accumulated elements in human hair and followed by As and Cu. The average single accumulative index of Hg, Pb, and Cd elements were 7.81, 6.75, and 4.20, respectively, which were consistent with the accumulative behavior of Hg, Pb, and Cd elements in soil, groundwater, and crops in the study area [34]. After excluding one adult with the highest content (268.57 mg/kg of Hg, 15.49 mg/kg of Pb, 10.09 mg/kg of Cd, and the comprehensive contamination index of 193.22), the toxic trace elements in the human hair of the remaining 21 adult males accumulated significantly with the single cumulative indexes as Hg of 18.7, Pb of 9.67, Cd of 6.09, and As of 4.13; followed by juvenile males, adult females, and juvenile females. The comprehensive cumulative index of the six toxic elements ranged from large to small were adult male (14.16), juvenile male (5.46), adult female (4.23), and juvenile female (4.03).

Table 5 shows that toxic trace elements in human hair in polluted areas were accumulated to various degrees. The cumulative rate of the Hg element in human hair of all the samples were 100% followed by Pb, Cd, and Cu, indicating that Hg is the most common accumulated element. The cumulative rate of Hg, Pb, Cd, and Cu were all 100% in the hair of adult males and followed by that in juvenile males. The results were consistent with the evaluation of Hg, Pb, and Cd elements in rivers, soils, vegetables, and crops in the previous study in this area [28,29,30,31,32,33,34,35,36,37,43].

Table 6 shows that the cumulative contribution rates of Hg, Pb, and Cd elements in human hair of different ages and both genders were the top three. The cumulative contribution rates of these three elements account for 83.13%, 81.47%, 78.36%, and 69.07% in adult males, adult females, juvenile males, and juvenile females, respectively. In the polluted area, the cumulative contribution rate of Hg in the hair of the adult males was the highest (45.19%), the cumulative contribution rate of Pb in the hair of the juvenile females was the highest (39.27%), and the cumulative contribution rate of Cd in the hair of the adult females was the highest (26.37%). This result revealed that the content of Pb in human hair has the most significant influence on adult females and juveniles, especially juvenile female. Moreover, Hg easily accumulated in adult bodies to a greater extent in males than in females; and Pb easily accumulated in juvenile bodies to a greater extent in females than in males.

### 3.3. Temporal Effects of Toxic Trace Elements in the Human Hair

The human hair samples from the eleven volunteers in polluted area and contrast area were collected in both 2019 and 2015, respectively (Table 7). The analysis results showed obvious differences in the toxic trace element contents and its variation trend in groups of both genders and different ages. The values were marked as negative or positive according to the results subtracted by the toxic trace element contents from the hair samples of the same volunteers in 2015 and 2019 (Table 7). The results showed almost a reduction trend among the contents of Hg and Cr in human hair from both the contrast area and the contaminated area. Comparing the results between 2019 and 2015, the increased toxic trace elements are mainly Cd (with growth rate of 100%) > Pb (with growth rate of 72.7%) > As (with growth rate of 63.6%) > Cu (with growth rate of 54.5%) > Hg (with growth rate of 9.1%), which indicates that the Cd content in human bodies increases significantly with a period of time, and Cd, Pb, As, and Cu become the main accumulated elements of human hair in the mining area, while the overall content of Hg decreases (only one human hair sample increases). This phenomenon is closely related to the recent government’s clear prohibition of beneficiation processes using the mercury flotation method and the reduced amount of Hg metal, but the reason for the higher arsenic content remains to be further studied.

Compared with the results of 2015 and 2019, no matter in the contrast area or the polluted area, the content of Hg and Cr in human hair were decreasing, and the contents of Pb, Cd, As, and Cu in human hair were increasing. Compared with the contrast area, the contents of Hg and Cr of the human hair decreased slightly whereas the content of Pb, Cd, As, and Cu of the human hair increased significantly in the polluted area. Generally speaking, the content of Pb, Cd, Cr, As, and Cu in human hair in the polluted area is still accumulating, and the sum of the single cumulative indexes of toxic trace elements (36.56) is 4.83 times higher than that in the contrast area (7.57). The toxic trace elements in human hair is obviously cumulated in 5 years and the average Pb and Cd contents in human hair in the polluted area increased by 0.28 and 0.10 mg/kg∙a^−1^.

### 3.4. Analysis of Environmental Pollution and Human Health

Toxic trace element pollution in human habitat environment is one of the important causes of human health problems [3,43,44,45]. The quantification of the relationship between environmental pollution and human health is difficult because of the latent, causal diversity, specificity, susceptibility, and uncertainty of the hazardous environmental pollution on human health [46]. Toxic trace elements in the environment could directly or indirectly affect the human body through soil, plants, animals, or air pollution and pose a threat to human health. Due to the differences in time and intensity of the human diet and exposure to polluted environment, some studies indicated the harmfulness of slow acting geochemical disasters manifests first in susceptible or vulnerable populations [47].

Wheat and seasonal vegetables in northern China are the main food sources for villagers in the study area. The average content of Hg in samples of wheat, vegetables, and drinking well water around village A is 45, 306, and 0.06 μg/kg, respectively. If calculated as 0.5 kg of flour, 0.5 kg of vegetables, and 1.0 kg of drinking water per person and day, the intake of Hg per person and day is 175.56 μg/kg, which is 17.56 times the international estimated daily total Hg threshold of 10 μg/day [48]. The estimation does not cover the daily amount of Hg inhaled by villagers from the atmospheric environment in the area. Obviously, environmental pollution has caused harm to the health of villagers. Among the 20 villagers inspected in the contaminated area in 2015, Hg and Pb in the hair of the villagers engaged in gold ore dressing and smelting activities were significantly higher than that of the long-term exposed population as well as the villagers from contrast area (Table 8). Fifteen of them experienced different health problems. Among the 9 villagers, whose comprehensive cumulative index of toxic trace elements was over 19, 8 of them were suffering from symptoms of dizziness, shortness of chest tightness, weakness, and high blood pressure. The high content of toxic trace elements in human hair has an obvious correlation with human health. The health of the people in the polluted area has been endangered mainly by Hg and Pb.

## 4. Discussion

As many studies have shown, hair can be a useful indicator for monitoring long-term exposure in contaminated areas [49,50,51,52]. The concentrations of Pb, Cu, Cd, As, and Hg in hair samples from the polluted area in this study were higher than the Chinese resident normal hair content of trace elements. Toxic trace elements can accumulate inside and outside the hair tissue during a long term of exposure [53]. In the process of hair growth, some metals in the blood and those in contact with soil particles or metals in the air will constantly integrate into the hair shaft [54]. Sulfur is evenly distributed in hair tissue, which provides a good platform for the absorption of elements. Mammalian hair is mainly composed of keratin, which contains a large number of sulfhydryl groups and can combine with a variety of metals. Elements in the environment are linked to sulfur atoms in S–H group of hair tissue and accumulate in the group [53]. Aluminum, arsenic, chromium, copper, and nickel are elements with high concentrations in external tissues such as hair [54]. The hair tissues of human beings are in direct and continuous contact with the floating dust in air because of their high activity around their habitations, such as farming, mining, smelting, and even indoor activities [55]. Dry and wet deposition of contaminated dust from heavy equipment activities is usually a more relevant way for human hair to absorb toxic elements. Consistent with our results, some studies have shown that the element levels in external tissues are relatively high in the hair of some animals [54,56,57,58,59]. Environmental factors, including metals in food, water, soil, and air, as well as age, gender, season, physiology, and health conditions, can affect the content of chemical elements in mammalian hair [52,55].

The assessment of the comprehensive cumulative pollution index (P_z_) revealed that males have significantly higher cumulative potentials of toxic trace elements than females. Many studies have shown that the stress response of different sexes to toxicity is quite different. In some mammals, including human beings, the bioaccumulation patterns of toxic elements usually exhibit gender differences [60,61,62,63,64,65,66]. Many factors can affect the bioaccumulation of essential elements between genders, such as gender-related occupational exposure, metabolic profile of metals, metal intake or absorption, activity of sex hormones, nutritional needs, or interactions between elements [64,67,68]. The gender with higher metabolic activity and long-term exposure may be more susceptible to specific, chemical-induced toxicity [69]. In the study area, males are the main group involved in mining and smelting, with high work intensity, fast physiological metabolism, and long exposure time. Therefore, they will be exposed for a longer time, consume more energy, and have higher metabolic activity [55,70]. However, the high metabolic rate of adult males indicates high intake of food, which may explain the increase of toxic trace element contents in the study area. In addition, different dietary habits may be an effective factor for toxic trace element bioaccumulation in different genders [71]. The results show that gender must be taken into account in order to accurately estimate the future environmental pollution based on the bioaccumulation of toxic elements in human development.

Compared with the previous studies on soil pollution in this study area, the single accumulation index of Hg, Pb, and Cd in human hair collected from the polluted area is higher, indicating the toxic elements may come from the soil [72]. The residents may be exposed to toxic trace elements in the polluted areas through a variety of ways, including inhalation of dust in the air, emissions from heavy equipment and vehicles, and ingestion of contaminated water or food and vegetables grown in soil. The concentrations of elements in different tissues of mammals, including humans, are directly proportional to their contents in food [73]. The residents in the study area usually have wheat and green vegetables for daily meals. Many kinds of toxic trace elements in the dust can be deposited on the surface of the vegetables and then absorbed into the vegetable tissue, thus increasing the content of these elements in the residents’ body in the study area.

The average concentrations of toxic elements in almost all human hair samples from the contaminated area were higher than those in the control area. In general, with the increase of the distance from the contaminated area, the element content in human hair will be decreased. Moreover, the toxic trace element contents in the human hair samples from the polluted area are mostly higher than that of the contrast area. Moreover, we observed that the single cumulative index of Cu and Cr in human hair was relatively low. In most cases, the increase of Cu content in the environment does not lead to the increase of Cu content in human tissues. Some studies have shown that the uptake and distribution of essential metals such as Cu and Zn in mammals (including humans) are physiologically regulated [74,75,76,77]. The possible reason is that the homeostasis mechanism can regulate the concentration of copper near the optimal value of metabolism in human body [78].

According to some previous studies on heavy metal accumulation in farmland soils in the Xiaoqinling gold mining area, Pb, Cu, Cd, and other trace elements showed significant accumulation trends in the soil from 2015 to 2018. This is the same as the accumulation trend of toxic trace elements in human hair in this study [79]. However, the concentration of toxic trace elements in human hair depends on many factors, such as gender, age, body shape, body weight, individual homeostasis mechanism, physiological state, differences in population structure, interaction between elements, changes in feeding patterns, pollution degree, pollutant concentration, and exposure time in exposure area [55,80,81]. It is still difficult to establish a reliable model for human health assessment only by using dietary habits, and more influencing factors need to be considered in future research.

## 5. Conclusions

In this study, hair samples were collected from residents of different genders, ages, and times (the years 2015 and 2019) in the Xiaoqinling gold mining area, Northwest China. The bioaccumulation effects of the six toxic trace elements in different ages and both genders were determined. Nonpolluted villages away from the mining industry concentration area with the same geological background were selected as the contrast area to explore the spatial variations of toxic trace element accumulation in the Xiaoqinling area. However, this work did not assess toxic trace element concentrations in other biological indicators (e.g., feces and urine) and other important organs (e.g., liver and kidney).This study recorded high levels of Hg, Pb, Cd, Cr, As, and Cu in the population aged from 2 to 77 in the Xiaoqinling gold mining area. The results showed that the average contents of Hg, Pb, and Cd in the hair of residents of different genders and ages in the contaminated area are higher than those in the contrast area. The average single cumulative indexes of Hg, Pb, Cd, and As among adult males were the highest in all groups, indicating the highest risk of toxic element concentration in the bodies of adult males. The average cumulative contribution rate of Hg, Pb, and Cd in hair of all the groups in the polluted area ranked among the top three, accounting for 78% of the total contribution rate. Therefore, Hg, Pb, and Cd pollution in the study area are considered to be the dominant factors threatening the health of residents. However, the content of Hg, Cr, and Cu in the hair of humans who directly engaged in mining and mineral processing were significantly higher than that of nonmining practitioners in polluted areas and residents in contrast area. This result shows that mining and mineral processing are the main sources of toxic elements in human body in the polluted area. Compared the results of 2015 and 2019, the contents of Hg and Cr in human hair were decreased while the contents of Pb, Cd, and Cu were increased in both contrast and contaminated areas. Compared with the contrast area, the contents of Hg and Cr decreased slightly while Cd, As, and Cu increased notably in the human hair from the polluted area. The overall results indicated that the contents of Hg and Cr in the environment were decreasing while the contents of Pb, Cd, As, and Cu were still accumulating.

In conclusion, toxic trace elements in human hair from polluted areas in the Xiaoqinling gold mining area were still accumulating, causing potential human health risk associated with the environmental pollution in gold mining areas. Therefore, local environmental management departments still need to continue to manage and control wastewater, solid waste, and fume discharges from mining and mineral processing industries. For historically contaminated farmland soils, necessary land improvement techniques and crops with weak uptake of toxic elements should be adopted. Of course, residents of the area should also be educated on the health issues associated with the bioaccumulation of toxic trace elements in the human bodies.

## Figures and Tables

**Table 1:** The average contents of toxic trace elements in the human hair in the contrast area.

Sample Groups (Numbers)	Hg	Pb	Cd	Cr	As	Cu
	mg/kg					
Adult males (5)	0.14	12.46	0.11	0.55	0.24	9.05
Adult female (6)	0.14	8.96	0.17	0.56	0.51	9.69
Juvenile male (3)	0.16	18.47	0.32	0.59	0.39	8.69
Juvenile female (2)	0.29	28.95	0.5	0.43	0.32	10.07
Standard deviation	0.07	8.11	0.16	0.06	0.10	0.54
Chinese resident normal hair content ^1^	0.77	6.60	0.29	1.21	0.68	11.30

^1.^ Standards from Chinese Trace Elements Scientific Society (H/ZWY03-2005, H/ZWY01-2007).

**Table 2:** The average contents of toxic trace elements in human hair of different ages in the polluted area.

Samples in Different Ages (Numbers)	Hg	Pb	Cd	Cr	As	Cu
	mg/kg					
2–9 (10)	1.098	193.92	1.335	0.452	1.075	78.197
10–17 (7)	0.417	57.957	0.646	0.471	0.407	11.136
18–29 (4)	0.828	74.325	0.945	0.563	0.286	43.643
30–39 (9)	0.811	78.456	0.354	0.532	0.841	13.366
40–49 (13)	3.492	77.248	0.844	0.471	0.431	14.565
50–59 (15)	0.546	80.773	0.715	0.571	0.756	14.816
60–77 (7)	1.207	94.129	0.813	0.516	0.500	12.683
Standard deviation	0.98	42.08	0.28	0.04	0.27	23.48

**Table 3:** The single cumulative index of toxic trace elements in human hair.

Sample Groups (Numbers)	Hg	Pb	Cd	Cr	As	Cu
	mg/kg					
Adult males in contrast area (5)	0.14	12.46	0.11	0.55	0.24	9.05
Adult males in polluted area (21)	2.63	120.44	0.67	0.63	0.99	15.59
The single cumulative indexes of adult males	18.78	9.67	6.09	1.15	4.13	1.72
Adult females in contrast area (6)	0.14	8.96	0.17	0.56	0.51	9.69
Adult females in polluted area (26)	0.57	47.25	0.76	0.44	0.28	17.40
The single cumulative indexes of adult females	4.07	5.27	4.47	0.79	0.55	1.80
Juvenile males in contrast area (3)	0.16	18.47	0.32	0.59	0.39	8.69
Juvenile males in polluted area (9)	1.01	125.02	1.39	0.49	1.01	11.97
The single cumulative indexes of Juvenile males	6.31	6.77	4.34	0.83	2.59	1.38
Juvenile females in contrast area (2)	0.29	28.95	0.35	0.43	0.32	10.07
Juvenile females in polluted area (8)	0.60	152.46	0.67	0.42	0.56	11.49
The single cumulative indexes of Juvenile females	2.07	5.27	1.91	0.98	1.75	1.14
Standard deviation	5.45	62.42	2.23	0.26	1.24	7.91

**Table 4:** The average single cumulative pollution indexes and average comprehensive cumulative pollution indexes of toxic trace elements in human hair in the polluted area.

Sample Groups (Numbers)	The Single Cumulative Index (P_i_)						The Comprehensive Cumulative Pollution Index (P_z_)
	Hg	Pb	Cd	Cr	As	Cu	
Adult males (21)	18.79	9.67	6.09	1.15	4.13	1.72	14.16
Adult females (26)	4.07	5.27	4.47	0.79	0.55	1.80	4.23
Juvenile males (9)	6.31	6.77	4.34	0.83	2.59	1.38	5.46
Juvenile females (8)	2.07	5.27	1.91	0.98	1.75	1.14	4.03
Average values	7.81	6.75	4.20	0.94	2.23	1.51	6.97

**Table 5:** The cumulative rate of toxic trace elements in human hair in the polluted area.

Sample Groups (Numbers)	Hg	Pb	Cd	Cr	As	Cu
	%					
Adult males (21)	100.0	100.0	100.0	68.2	86.4	100.0
Adult females (26)	100.0	84.6	80.8	11.5	Unaccumulated	73.1
Juvenile males (9)	100.0	100.0	88.9	11.1	77.8	88.9
Juvenile females (8)	100.0	75.0	62.5	12.5	62.5	50.0

**Table 6:** The cumulative contribution rate of toxic trace elements in human hair from different groups in the polluted area.

Sample Groups (Numbers)	Hg	Pb	Cd	Cr	As	Cu
	%					
Adult males (21)	45.19	23.28	14.66	2.77	9.97	4.14
Adult females (26)	24.01	31.09	26.37	4.66	3.24	10.62
Juvenile males (9)	28.43	30.45	19.48	3.78	11.65	6.21
Juvenile females (8)	15.42	39.27	14.38	7.30	13.11	10.51
Average values	28.26	31.02	18.72	4.63	9.49	7.87

**Table 7:** The variation of toxic trace elements in human hair from 2015 to 2019.

Samples	Hg	Pb	Cd	Cr	As	Cu
	mg/kg					
F_1d_	−0.15	10.00	2.60	−1.25	−1.01	−0.11
F_1d_ female	−0.19	5.60	2.60	−1.94	0.05	−1.07
F_1_ female	−0.84	33.01	0.73	−0.71	23.00	5.90
F_2_	−0.12	13.30	1.60	−1.27	−0.11	2.11
F_3_	−0.29	−9.00	5.80	−1.24	−0.39	1.30
F_4_ female	−1.19	8.60	11.60	−0.80	31.00	2.70
F_5_	−0.53	−22.10	2.80	−1.15	19.00	−0.31
F_6_	−0.67	−19.00	0.49	−0.88	0.24	−3.70
F_7_ female	−0.99	118.80	4.80	−1.09	23.00	7.20
F_8_	30.96	71.00	1.05	−1.16	0.09	20.10
F_9_	−0.41	119.30	9.00	−4.79	29.00	1.20
The growth rate R_g_ (%)	9.10	72.70	100.00	0.00	63.60	54.50

F_1d_ and F_1d_ female samples were collected from the contrast area, while the others were collected from the polluted area.

**Table 8:** The comparison of the toxic trace elements in human hair from the volunteers working on mining processing and the contrast area.

Sample Groups	Hg	Pb	Cr	Cu
	mg/kg			
Working on mining and mineral processing	3.54	88.57	1.51	11.32
Live in the polluted area	1.38	51.56	2.21	10.91
Live in the contrast area	0.29	3.03	2.03	6.48

## Data Availability

The data presented in this study are available on request from the corresponding author. The data are not publicly available due to the Data Management Policy of China Geological Survey.
